# Commercial and Custom Quartz Tuning Forks for Quartz Enhanced Photoacoustic Spectroscopy: Stability under Humidity Variation

**DOI:** 10.3390/s23063135

**Published:** 2023-03-15

**Authors:** Diba Ayache, Roman Rousseau, Elena Kniazeva, Julien Charensol, Tarek Seoudi, Michael Bahriz, Fares Gouzi, Vincenzo Spagnolo, Aurore Vicet

**Affiliations:** 1 IES, CNRS, University Montpellier, 34095 Montpellier, France; diba.ayache@ies.univ-montp2.fr (D.A.);; 2 PolySense Laboratory, Università Degli Studi di Bari Aldo Moro e Politecnico di Bari, 70126 Bari, Italy; 3 PhyMedExp, INSERM, CNRS, CHRU, University of Montpellier, 34000 Montpellier, France

**Keywords:** sensing, spectroscopy, Quartz tuning forks, QEPAS

## Abstract

This work investigates the behavior of commercial and custom Quartz tuning forkss (QTF) under humidity variations. The QTFs were placed inside a humidity chamber and the parameters were studied with a setup to record the resonance frequency and quality factor by resonance tracking. The variations of these parameters that led to a 1% theoretical error on the Quartz Enhanced Photoacoustic Spectroscopy (QEPAS) signal were defined. At a controlled level of humidity, the commercial and custom QTFs present similar results. Therefore, commercial QTFs appear to be a very good candidates for QEPAS as they are also affordable and small. When the humidity increases from 30 to 90 %RH, the variations in the custom QTFs’ parameters remain suitable, while commercial QTFs show unpredictable behavior.

## 1. Introduction

First introduced in 2002 by Kosterev et al. [1], Quartz Enhanced Photoacoustic Spectroscopy (QEPAS) has been widely employed for gas sensing applications. This technique relies on photoacoustic principles but exploits a Quartz tuning forks (QTF) as an optoacoustic transducer. The standard QTF is a low-cost piezoelectric element component used in the watch industry. Its fundamental resonance frequency draws a sharp and intense Lorentzian shaped line around 32.7 kHz. Despite the fact that the sensitivity of QEPAS has been proven through numerous sensing demonstrations, the original frequency is not the optimal one for photoacoustic sensing: some molecular deexcitations need to be addressed with lower frequencies [2]. In addition, the time constant associated with the standard QTF complete deexcitation [3] is relatively high compared to the commonly used lock-in amplifiers (LIA) time constants in photoacoustic sensing, which implies an out-of-equilibrium detection scheme of the QTF. However, its high-quality factor (Q) (Q~100,000 in vacuum and Q~8000 in atmospheric pressure) and its quadrupole geometry renders QEPAS nearly immune to ambient noises compared to the standard microphones used in classical photoacoustic spectroscopy (PAS). The detection scheme includes a laser source, wavelength modulated at the QTF resonance frequency (or its sub-harmonics) and slowly tuned across one or several gas absorption lines. For better matching between the relaxation time of the molecules and the QTF resonance frequency, water can be added to the gas mixture: for some slow-relaxing molecules, such as carbon monoxide [4,5], carbon dioxide [6] and nitric oxide [7], humidity works as a promotor of the photoacoustic effect by reducing the target molecules’ overall relaxation time. An alternative solution would be the reduction of the modulation frequency by employing custom QTFs that resonate at frequencies down to a few kHz. Indeed, several research groups have been working on the development of custom QTFs. The advantages of the newly introduced QTFs also rely on their shape as, not only do they operate at lower resonance frequencies, but they are also characterized by larger prongs spacing (up to 1.5 mm); this makes the alignment of the laser more comfortable and avoids the arising of photothermal perturbations during the measurements, especially at long wavelengths. A French team in ONERA (Paris, France) is focused on the development of a large QTF with very high quality factors [8,9], while an Italian team in Polysense (Bari, Italy) has realized custom QTFs dedicated to terahertz (THz) gas sensing [10], with larger prong spacing (1 mm) well adapted to long wavelength quantum cascade lasers (QCLs). In Bari, the first custom QTFs were characterized by resonance frequencies as low as 2.8 kHz and quality factors varying between 7000 and 37,000 at atmospheric pressure. These QTFs were designed by etching a z-cut quartz wafer using photolithographic techniques, and electrical contacts were made with chromium and gold [11,12]. The first application was dedicated to the detection of methanol with a THz laser source using a QTF with a resonance frequency equal to 4245 Hz and a quality factor of 76,300 at 10 Torr [10].

Subsequently, the geometry was adapted, targeting different frequencies [13]. The influence of the prongs’ length and thickness on the frequency and quality factor of the QTF were investigated. Different shapes were tested in this second generation. By adding grooves on each side of the QTF, or by designing T-shaped prongs, a good compromise between low resonance frequencies, high quality factors and a small resistance has been found. The so-called “T-shape” QTF with a resonance frequency around 12 kHz and a Q-factor~14,000 at atmospheric pressure is now commercialized in an acoustic detection module produced by Thorlabs [14].

While it is almost immune to acoustic noises, QEPAS is sensitive to environmental parameter variations. The temperature, pressure and humidity can modify the QTF properties, generating a shift in the resonance frequency (*f*_0_) and/or decreasing the quality factor (Q) [15]. The variations observed in QEPAS are expected to be due to concentration and gas density changes in the medium; however, as the measured output signal strongly depends on these exterior parameters, it appears important to either:

- Follow the *f*_0_ and Q evolution in real time and operate a feedback correction on the optical and electrical setup, i.e., on the modulation of the laser and the LIA parameters.
- Reduce their influence by maintaining their variations in a given controlled range.

In this paper, we propose to evaluate the stability of commercial and custom QTF parameters under humidity variations. As part of a collaboration with Polysense, we were able to build this study between a standard QTF and four of their designed QTFs.

## 2. Important QTF Parameters to Evaluate in QEPAS Sensing: Estimation of the Required Accuracy on *f*_0_ and Q

Most industrial gas sensors assure an accuracy of a few percent on the displayed value (signal amplitude, concentration). We have decided to follow this criterion to define an acceptable relative error of 1% on the QEPAS signal (Equation (1)). Therefore, the first step is to calculate the required accuracy for the *f*_0_ and Q to not exceed this 1% error.

(1) $$\frac{\Delta S}{S\left(f_{0}\right)}<\;0.01.$$

We consider here a QEPAS signal S modelized by a Lorentzian lineshape, centered on *f*_0_ as the resonant frequency, describing the frequency response of a typical QTF. It corresponds to the well-known Butterworth-Von Dyke (BVD) electrical model of the component. The squared QEPAS signal is proportional to the squared admittance of the QTF, representing a Lorentzian function [16]. This shape does not depend on the QEPAS sensing technique, harmonic detection by wavelength modulation (1f or 2f) or amplitude modulation. It represents the electrical response of the QTF to an acoustic excitation. We have:

(2) $$S^{2}\left(f,\;Q\right)=C\frac{Q^{2}}{1+{\left(\frac{2Q\left(f-f_{0}\right)}{f_{0}}\right)}^{2}}\;$$

where C is a constant taking into account the transimpedance gain and the BVD model parameters, which are included in the *f_0_* and Q as well. This 1% amplitude error can be converted in an error on the measured frequency $\Delta f_{0}$.

Isolating f in (2) gives:

(3) $$f\left(S\right)=\pm \frac{f_{0}}{2Q}\;\sqrt{\frac{{\mathrm{CQ}}^{2}}{S^{2}}-1}{+f}_{0}\;.$$

Using (3), and noting that:

(4) $$S^{2}\left(f_{0}\right){=\mathrm{CQ}}^{2}\;$$

one can find the frequency error, and its numerical value for a commercial QTF with *f*_0_ = 32 kHz and typical Q of 8000:

(5) $${\Delta f}_{0}=\left|f_{0}-f\left(S-\Delta S\right)\right|=\frac{f_{0}}{2Q}\;\sqrt{\frac{1}{{\left(1-\frac{S\left(f_{0}\right)}{\Delta S}\right)}^{2}}-1}\;$$

(6) $${\Delta f}_{0}=\frac{{32\times 10}^{3}}{2\times 8000}\;\sqrt{\frac{1}{{\left(1-0.01\right)}^{2}}-1\;}=0.28\;\mathrm{Hz}.$$

**Figure 1:**
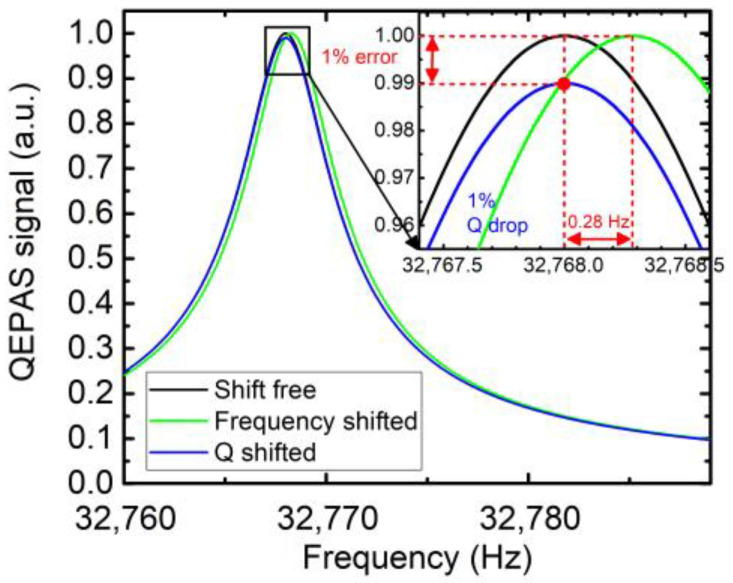
The frequency response of the shift-free QEPAS signal (black) is represented. It is a Lorentzian curve centered at 32,768 Hz, having a quality factor of 8000. The QEPAS signal reaches a maximum value at *f = f*_0_. The frequency response is also shown for a frequency shift of 0.28 Hz (green) and Q-factor reduction of 80 (blue). The two curves intersect (red dot) at *f = f*_0_, corresponding to a 1% QEPAS signal error as calculated.

This variation in the frequency corresponds to the green curve in Figure 1. This amplitude error can be converted in an error on the quality factor as well. At *f* = *f*_0_, the QEPAS signal has a linear relationship with Q, thus the error is easily obtained:

(7) $$\frac{\Delta Q}{Q}=\frac{\Delta S}{S\left(f_{0}\right)}\to \;\Delta Q=0.01Q=80,$$

it is represented by the blue curve in Figure 1. From these calculations, it can be concluded that a frequency shift of 0.28 Hz or a Q variation of 80 will lead to a 1% relative error on the QEPAS signal.

In this study, we will consider ten QTFs: two under the “AV-08” reference, two under the “T1-08” reference and six under the “commercial QTF” reference. “AV-08” and “T1-08” are custom QTFs realized by Polysense. The commercial QTFs come from Fox Electronics (NC38LF).

**Figure 2:**
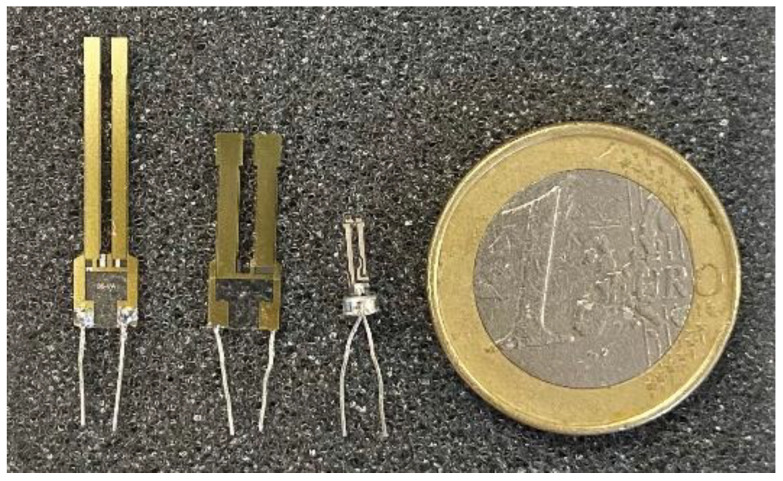
From left to right a sample of each QTF is presented: AV-08, T1-08, commercial.

For each tested QTF, an estimation of Δ*f*_0_ and ΔQ in relation to a 1% signal variation is calculated. Table 1 summarizes the theoretical calculated values and describes the QTFs considered in this study. Figure 2 presents the different tested QTFs.

## 3. Experimental Setup and Results

When an external perturbation is brought to an oscillating device, it oscillates according to the frequency imposed by the perturbation. When this perturbation is stopped, the oscillation returns to the inner frequency of the device: it represents the relaxation phase. In the presented experimental setup, a sinewave of frequency *f*_exc_ and amplitude V_exc_ was generated using a waveform generator (Tektronix AFG 1022).

The measurement was performed following different steps:

1. The sinewave was used to electrically excite the QTF (during t_exc_).
2. The excitation was stopped.
3. The relaxation signal was recorded on a LabVIEW program.

This setup was thoroughly described and explained in [17]. The results of the performed study are exploited in this manuscript.

The frequency *f*_exc_ used for the exciting sinewave was also defined as a reference frequency for the LIA (Zurich Instruments MFLI). The signal recorded from the QTF was amplified with a homemade transimpedance amplifier and demodulated at *f*_exc_ by the LIA. The collected signal has an exponential decay shape. Its period and envelop are, respectively, described by Equations (8) and (9). While the former gives information about the instantaneous frequency of the QTF, the latter gives access to its quality factor.

(8) $$T=\frac{1}{{{|f}_{0}-f_{\mathrm{exc}}|}^{\prime }}$$

(9) $$V_{\mathrm{out}}{(t)=V}_{\mathrm{exc}}{\times \;e}^{\frac{-{\pi \mathrm{tf}}_{0}}{Q}}.\;$$

For instance, *f_exc_* must be chosen to be close enough to *f*_0_ to obtain a good accuracy on the demodulated signal, but far enough to avoid *f*_0_ *= f*_exc_, leading to huge values of T. In parallel, V_exc_ must be chosen to be big enough to avoid uncertainty on the demodulated signal, but small enough to avoid the saturation of the LIA.

To determine the best experimental conditions, the effect of the sinewave parameters (*f*_exc_ and V_exc_) on the QTF parameters (*f*_0_ and Q) was measured.

**Figure 3:**
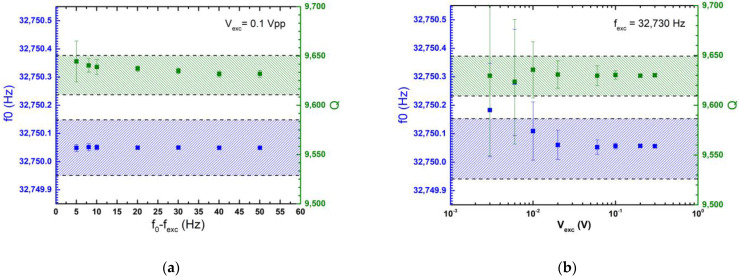
Measured QTF parameters as a function of the excitation frequency (**a**) and the excitation amplitude (**b**). The hatched areas correspond to the target accuracy and the error bars to the standard deviation. The excitation time t_exc_ is set to 200 ms to ensure the QTF is at steady state before the onset of the relaxation.

The results of the commercial QTF are reported in Figure 3. For each data point, the demodulated signal is recorded for 60 s at a rate of 1 Hz. The mean value and the standard deviation are calculated. The acceptable error intervals for *f*_0_ and Q calculated in the previous section are represented by shaded areas. In Figure 3a, V_exc_ is arbitrary fixed to 0.1 V_pp_ and *f_exc_* is chosen to have |*f*_0_ − *f*_exc_| variation between 5 and 50 Hz. In Figure 3b, |*f*_0_ − *f*_exc_| = 20 Hz and V_exc_ ranges between 3 and 300 mV_pp_.

On one hand, for variations of |*f*_0_ − *f*_exc_| in the range of [5, 50] Hz, *f*_0_ showed a nearly flat response. These observations assure that the measurement is robust and will not be affected by a sudden shift in *f*_0_. On the other hand, for low amplitudes, the large error bars illustrate the random error caused by a poor signal-to-noise ratio (SNR). When the excitation amplitude increases, the dispersion decreases, and the mean values quickly converge. This preliminary study was useful for quantifying the errors in the characterization setup.

The same study was conducted for each QTF described in this paper. Finally, according to the results, |*f*_0_ − *f*_exc_|was chosen to be equal to 20 Hz, 2 Hz and 3 Hz for the commercial QTF, AV-08 group and T1-08 group, respectively. V_exc_ was chosen to be equal to 100 mV_pp_ in all of the experiments.

Once these parameters are fixed, one can move on to the measurement and analysis of the shifts in *f*_0_ and Q under the humidity variation.

The behavior of the QTF parameters under humidity variation was assessed with a benchtop humidity chamber (ESPEC SH-242). The temperature in the chamber was set to 22 °C. The humidity was changed from 30% to 90% RH (percentage of relative humidity), with an increasing step of 10% occurring every 20 min. To confirm the observations and ensure their repeatability, a step to 50% and 30 %RH was added after the 90 %RH step. The humidity steps were long due to the response time of the humidity chamber, which was in the range of a few minutes.

**Figure 4:**
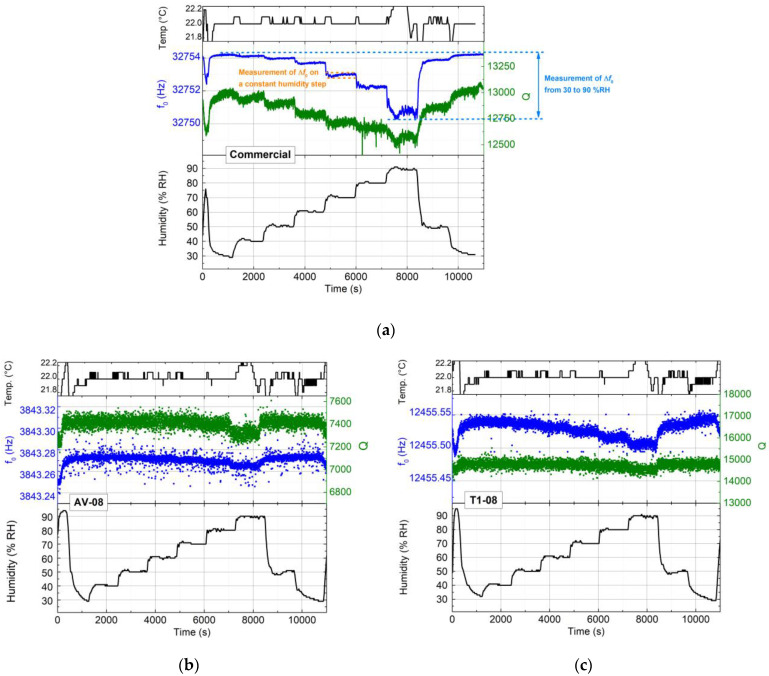
Frequency and quality factor measurement for different humidity steps with a fixed temperature of 22 °C. Humidity and temperature data were collected from the humidity chamber software. Variation of *f*_0_ and Q were measured with a LabVIEW program. (**a**) commercial QTF, (**b**) AV-08 and (**c**) T1-08. The time constant of the LIA was set to 1 ms for proper BF demodulation. The study of *f*_0_ and Q variation for different steps of humidity was performed in two conditions described in blue and orange dashed lines (Figure 4a).

The resonance frequency of each QTF, its quality factor, as well as the temperature and the different humidity steps were recorded and are presented in Figure 4.

The study of the variation in the QTF parameters was made in two steps:

- Investigating the **deviation of *f*_0_ and Q during a stabilized value of humidity** (arbitrary measured for 70 %RH) as it could be the case in a QEPAS experiment with controlled humidity.
- **The difference of the mean values of *f*_0_ and Q between 30% and 90 %RH** is investigated considering the case of a real-life measurement, where the humidity would increase rapidly.

Figure 4a describes the variation in the commercial QTF parameters. It shows a shift of approximately 2 Hz when varying the humidity from 30 to 80 %RH and about 1.5 Hz between 80 and 90 %RH, which represents a maximum shift of approximately 3.5 Hz between the lowest and highest value of humidity. At a constant value of humidity, the *f*_0_ variations were very small: ~0.025 Hz. The same investigations were realized for the custom QTFs, and the results are summarized in Table 2. For each QTF, the largest shift of *f*_0_ occurred at 90 %RH. The variation in *f*_0_ when moving from 80 to 90 %RH was approximately equal to the shift that occurred when the humidity was increased from 30 to 80 %RH. It is also at 90 %RH that the largest error in the temperature was observed. This error, approximately equal to ±0.2 °C, occurred due to the difficulties of keeping this humidity value stable.

In Table 2, the results are presented for a commercial QTF and for each group of custom QTFs. Several measurements were performed on the commercial QTFs, and the mean values were extracted from these experiments, including six QTFs.

## 4. Discussion

Usually, before any QEPAS measurement, a frequency sweep can be realized to estimate the exact resonance frequency of the QTF. Simultaneously, the measured frequency is used as the laser modulation frequency and demodulation frequency in the LIA. This frequency is also used as a reference in the demodulation of the collected signal. In controlled experimental conditions, there is no reason to observe significative changes between the measured frequency and the one used as a reference by the LIA. However, in real-life applications, the humidity, temperature and gas flow do not always remain constant. For example, in environmental gas monitoring, the humidity depends on the weather conditions. In breath analysis, the concentration of water is considered very high: close to 5%, i.e., 90 %RH, and can vary from one subject to another. The effect of the temperature on the QTF is well-known and specified by manufacturers. The frequency evolves in a parabolic shape when the temperature increases [17]. The efficiency of the characterization technique used in this paper was first evaluated through temperature variation. It appeared to be a straightforward way to compare the obtained results to a certified reference. The effect of the humidity was questioned when the measurements started to use QTFs in atmospheric conditions, but the literature lacks information on the effect of that parameter. In the literature, the effect of the humidity on the resonance frequency was investigated when using QTFs as humidity sensors as they can be very sensitive to mass changes [18]. Hygroscopic coating was added onto the QTFs’ prongs, and the effect of the different layers was investigated. The variations were measured with an oscillating circuit: the shift of the frequency is negative when the humidity increases and more pronounced for a thicker coating as the surface for water deposition is increased.

In QEPAS measurements, humidity can be useful to enhance the photoacoustic effect, but at the same time, can be a source of interferences and error. In this paper, the presented results are interpreted on the basis of an acceptable error of 1% on the QEPAS signal. The calculated variation of *f*_0_ leading this 1% error were equal to 0.28, 0.036 and 0.06 Hz, respectively, for the commercial, AV-08 and T1-08 groups.

At a constant value of humidity, i.e., by keeping the humidity level stable during the experiment, both the commercial and custom QTFs presented non-significative variations in their resonance frequency, showing that the characterization setup is stable and accurate. In fact, there was a factor of approximately 0.1 between the measured variations of *f*_0_ and the calculated values leading to 1% of error for each group of QTFs.

The comparison of the frequency variations between 30 and 90 %RH showed a difference in behavior between the custom QTFs and the commercial ones. Several commercial QTFs were tested; the dispersion of the data shows unpredictable variations in high humidity conditions (80–90 %RH), whereas both groups of custom QTF presented sustainable variation in the same conditions. For the commercial QTFs, the variation of *f*_0_, ranging between 0.5 and 3 Hz, was above the acceptable defined values. In this comparison, the AV-08 group presented more stable results than the T1-08. The measured values for AV-08 (resp., for T1-08) were approximately 0.0055 (resp., 0.05) and were five times (resp., 1.2 times) smaller than the calculated values.

Even if they were not especially designed for photoacoustic uses, the commercial QTFs presented very comparable QEPAS performances to the custom QTFs, as long as the humidity was stabilized during the measurement. Therefore, commercial QTFs remain a cheaper, smaller and efficient option for gas sensing. Nevertheless, in some applications, such as breath analysis, the relative humidity can reach 90% at the end of the exhalation, implying a large increase in the humidity in the sensor that usually operates at room humidity. The results obtained through these measurements showed more that the custom QTFs gave more reliable performances in this regard, while the commercial QTFs’ behavior can be unpredictable.

Most field sensors do not monitor only one type of information, such as the gas concentration, but often integrate temperature and humidity probes. The data of these environmental parameters can be displayed in real-time and eventually be used for post-process, data analysis and correlations, as well as for the real-time compensation of these parameters onto the output signal. The irregular variation of *f*_0_ leads us to focus our concern of this crucial parameter, which was taken into consideration at different stages in the experiments. However, the inconsistent variations in the quality factor are more complex to describe. For the commercial QTFs, the variation of Q from 30 to 90 %RH might be a cause of the instability in the system, while they are acceptable at a constant humidity value. For the custom QTFs, no trends can be extracted in the measurement of Q.

Different hypotheses can be proposed to explain the *f*_0_ and Q variations:

- During the measurements presented in Figure 4, the QTF was connected to a metallic support and the support was in mechanical contact with the metallic surface of the humidity chamber. The mechanical vibrations of the support, as well as the air flow generated by the fan present inside the chamber, are possibly the cause of this “degradation” observed in the Q. It was even more pronounced for the custom QTF due to their longer length and thinner thickness. The measurements presented in Figure 3 were performed at a constant humidity level. These measurements were performed before launching the machine, i.e., with the machine’s door open; thus, the conditions of the temperature and humidity inside the chamber are equal to the ambient ones. The dispersion observed in this figure for the chosen parameters (|*f*_0_ − *f*_exc_| = 20 Hz and V_exc_ = 100 mV_pp_) is less significant than that reported during the measurement performed with humidity variations.
- The QTFs were exposed to high levels of humidity. The experiments presented in this paper were conducted at least two times. The surface of the QTFs was observed with a microscope. However, with a magnification down to 100x, no physical degradations of the QTFs were noticed.
- In the investigation of the QTF as a humidity sensor [18], the presence of small water droplets settling on the surface of the prongs due to their roughness was considered. This added non-uniform mass may be the cause of the observed damping.

## 5. Conclusions

In this work, the evaluation of custom and commercial QTFs’ parameters, along with humidity variations, was presented. Our attention was focused on the variation in the resonance frequency of the transducer as the QEPAS signal strongly relies on that parameter. The performed measurement has shed light on the importance of controlling the humidity during spectroscopy and sensing experiments as it has a non-neglectable impact on the stability of this frequency. By the mean of the developed experimental setup, we were able to monitor the *f*_0_ and Q variations under temperature and humidity changes. In real-life applications, such as breath analysis, the regulation of these parameters’ effect on the measurements becomes crucial. The consistency of the QTFs especially designed for gas sensing was proven during the experiment, while the commercial QTFs displayed more instability at high values of humidity. While we are still uncertain of the origin of the high dispersion observed in the commercial QTF, one must consider the necessity to perform additional measurements on each group of QTF in order to build a statistic and to potentially observe a trend. Further measurements for a better understanding of the humidity effect may imply the use of several coatings on the commercial QTFs or the development of custom QTFs with dimensions that are closer to the commercial ones.

## Figures and Tables

**Table 1:** Considering a 1% error on the QEPAS signal to be acceptable, the Δ*f*_0_ and ΔQ values are calculated for samples AV-08, T1-08 and commercial QTF (NC38LF by Fox Electronics). Measurements are performed on the fundamental mode.

Ref QTF	*f*_0_ (Hz)	Q	Δ*f*_0_ (mHz)	ΔQ	Prong Length (mm)	Prong Width (mm)	Prong Spacing (mm)
AV-08	3800	7500	36	75	16.0	1.2	0.8
T1-08	12,450	15,000	60	150	Base: 7.0 Head: 2.4	Base: 1.4 Head: 2.0	0.8
Commercial	32,750	8000	280	80	~3.8	0.6	0.3

**Table 2:** Comparison of measured variation of *f*_0_ and Q to the related calculated variation leading to 1% error on the measured QEPAS signal. The measured variation of *f*_0_ and Q are obtained by varying the humidity between 30 and 90 %RH.

	Variation of *f*_0_ (Hz)			Variations of Q		
	Target	Experimental		Target	Experimental	
	Calculated for 1% Error	Inter-Step 30–90 %RH	Intra-Step 70 %RH	Calculated for 1% Error	Inter-Step 30–90 %RH	Intra-Step 70 %RH
Commercial QTF presented	0.280	3.5	0.025	80	391.5	22.7
Statistics on commercial QTF	0.280	1.105 ± 1.612	0.01 ± 0.01	80	365.7 ± 393.1	19.1 ± 2.8
QTF AV-08 A	0.036	0.0055	0.0035	75	10	100
QTF AV-08 B	0.036	0.0087	0.0058	75	86	60
QTF T1-08 A	0.060	0.052	0.0064	150	40	300
QTF T1-08 B	0.060	0.041	0.0042	150	381.7	250

## Data Availability

The data presented in this study are available on request from the corresponding author.

## References

[1] Kosterev A.A., Bakhirkin Y.A., Curl R.F., Tittel F. (2002). Quartz-Enhanced Photoacoustic Spectroscopy. Opt. Lett..

[2] Schilt S., Besson J.P., Thévenaz L. (2006). Near-Infrared Laser Photoacoustic Detection of Methane: The Impact of Molecular Relaxation. Appl. Phys. B Lasers Opt..

[3] Rousseau R., Ayache D., Trzpil W., Bahriz M., Vicet A. (2021). Passive Electrical Damping of a Quartz Tuning Fork as a Path to Fast Resonance Tracking in Qepas. Sensors.

[4] Maurin N., Rousseau R., Trzpil W., Aoust G., Hayot M., Mercier J., Bahriz M., Gouzi F., Vicet A. (2020). First Clinical Evaluation of a Quartz Enhanced Photo-Acoustic CO Sensor for Human Breath Analysis. Sens. Actuators B Chem..

[5] Sgobba F., Sampaolo A., Patimisco P., Giglio M., Menduni G., Ranieri A.C., Hoelzl C., Rossmadl H., Brehm C., Mackowiak V. (2022). Compact and Portable Quartz-Enhanced Photoacoustic Spectroscopy Sensor for Carbon Monoxide Environmental Monitoring in Urban Areas. Photoacoustics.

[6] Wysocki G., Kosterev A.A., Tittel F.K. (2006). Influence of Molecular Relaxation Dynamics on Quartz-Enhanced Photoacoustic Detection of CO2 at λ = 2 Μm. Appl. Phys. B Lasers Opt..

[7] Dong L., Spagnolo V., Lewicki R., Tittel F.K. (2011). Ppb-Level Detection of Nitric Oxide Using an External Cavity Quantum Cascade Laser Based QEPAS Sensor. Opt. Express.

[8] Aoust G. (2016). Développements de Sources Infrarouges et de Résonateurs En Quartz Pour La Spectroscopie Photoacoustique. Ph.D. Thesis.

[9] Duquesnoy M., Aoust G., Melkonian J.M., Lévy R., Raybaut M., Godard A. (2019). Quartz Enhanced Photoacoustic Spectroscopy Based on a Custom Quartz Tuning Fork. Sensors.

[10] Borri S., Patimisco P., Sampaolo A., Beere H.E., Ritchie D.A., Vitiello M.S., Scamarcio G., Spagnolo V. (2013). Terahertz Quartz Enhanced Photo-Acoustic Sensor. Appl. Phys. Lett..

[11] Lee S., Lee J.Y., Park T.S. (2001). Fabrication of SMD 32.768 KHz Tuning Fork-Type Crystals: Photolithography and Selective Etching of an Array of Quartz Tuning Fork Resonators. Werkstoffe Korrosion.

[12] Patimisco P., Borri S., Sampaolo A., Beere H.E., Ritchie D.A., Vitiello M.S., Scamarcio G., Spagnolo V. (2014). A Quartz Enhanced Photo-Acoustic Gas Sensor Based on a Custom Tuning Fork and a Terahertz Quantum Cascade Laser. Analyst.

[13] Patimisco P., Sampaolo A., Giglio M., dello Russo S., Mackowiak V., Rossmadl H., Cable A., Tittel F.K., Spagnolo V. (2019). Tuning Forks with Optimized Geometries for Quartz-Enhanced Photoacoustic Spectroscopy. Opt. Express.

[14] Thorlabs. https://www.Thorlabs.Com/Newgrouppage9.Cfm?Objectgroup_id=11241&pn=ADM01.

[15] Patimisco P., Sampaolo A., Dong L., Giglio M., Scamarcio G., Tittel F.K., Spagnolo V. (2016). Analysis of the Electro-Elastic Properties of Custom Quartz Tuning Forks for Optoacoustic Gas Sensing. Sens. Actuators B Chem..

[16] Friedt J.-M., Carry É. (2007). Introduction to the Quartz Tuning Fork. Am. J. Phys..

[17] Rousseau R., Maurin N., Trzpil W., Bahriz M., Vicet A. (2019). Quartz Tuning Fork Resonance Tracking and Application in Quartz Enhanced Photoacoustics Spectroscopy. Sensors.

[18] Carullo A., Vallan A., Afify A.S., Tulliani J.M. Development of a Fast Humidity Sensor Based on Quartz Tuning Fork. Proceedings of the Conference Record–IEEE Instrumentation and Measurement Technology Conference.

